# TDTHub, a web server tool for the analysis of transcription factor binding sites in plants

**DOI:** 10.1111/tpj.15873

**Published:** 2022-07-01

**Authors:** Joaquín Grau, José M. Franco‐Zorrilla

**Affiliations:** ^1^ Department of Plant Molecular Genetics Centro Nacional de Biotecnología CNB‐CSIC, C/Darwin 3 28049 Madrid Spain

**Keywords:** transcription factor (TF), plants, TF binding sites (TFBSs), gene regulation, bioinformatics, comparative genomics

## Abstract

Transcriptional regulation underlies most developmental programs and physiological responses to environmental changes in plants. Transcription factors (TFs) play a key role in the regulation of gene expression by binding specifically to short DNA sequences in the regulatory regions of genes: the TF binding sites (TFBSs). In recent years, several bioinformatic tools have been developed to detect TFBSs in candidate genes, either by *de novo* prediction or by directly mapping experimentally known TFBSs. However, most of these tools contain information for only a few species or require multi‐step procedures, and are not always intuitive for non‐experienced researchers. Here we present TFBS‐Discovery Tool Hub (TDTHub), a web server for quick and intuitive studies of transcriptional regulation in plants. TDTHub uses pre‐computed TFBSs in 40 plant species and allows the choice of two mapping algorithms, providing a higher versatility. Besides the main TFBS enrichment tool, TDTHub includes additional tools to assist in the analysis and visualization of data. In order to demonstrate the effectiveness of TDTHub, we analyzed the transcriptional regulation of the anthocyanin biosynthesis pathway. We also analyzed the transcriptional cascades in response to jasmonate and wounding in Arabidopsis and tomato (*Solanum lycopersicum*), respectively. In these studies, TDTHub helped to verify the most relevant TF nodes and to propose new ones with a prominent role in these pathways. TDTHub is available at http://acrab.cnb.csic.es/TDTHub/, and it will be periodically upgraded and expanded for new species and gene annotations.

## Significance Statement

Transcription factors (TFs) are the main players during regulation of gene expression through recognition of cognate DNA motifs, the TF binding sites (TFBSs). Most bioinformatic tools to detect TFBSs are restricted to only a few species or require multi‐step procedures. We present TDTHub, a web server for quick and intuitive studies of transcriptional regulation in plants. TDTHub uses pre‐computed TFBSs in 40 species and includes additional tools to help in the analysis and visualization of data.

## INTRODUCTION

In a changing environment, plants need to adapt their growth and development for survival. These adaptive responses mostly involve transcriptional reprogramming, which is tightly controlled by sequence‐specific transcription factors (TFs). TFs are responsible for the transcriptional activation or repression of target genes by recognizing specific, short DNA sequences (6–12 base pairs [bp]) in their regulatory regions, referred to as TF binding sites (TFBSs). Transcriptional regulation is a quite complex process that involves the activity of hundreds or even thousands of TFs that can act either alone or cooperatively to regulate the expression of a multitude of genes. It has been estimated that the average number of target genes for each TF is 2785 and the average number of regulatory TFs for each target gene is 25.7 (Jones & Vandepoele, 2020). Therefore, transcriptional regulation is a combinatorial result of *cis*‐ (i.e., TFBSs in regulatory regions) and *trans*‐acting – the TFs – elements.

Several *in vivo* and *in vitro* experimental approaches have contributed to describe hundreds of TFBSs (Franco‐Zorrilla & Solano, 2017). Among *in vivo* strategies, chromatin immunoprecipitation followed by sequencing (ChIP‐seq), DNase I treatment followed by sequencing (Pajoro et al., 2014; Sullivan et al., 2014), and the assay for transposase‐accessible chromatin followed by sequencing (Buenrostro et al., 2013; Maher et al., 2018) have been utilized to identify target genes or accessible chromatin regions, but direct identification of the TFBSs has been more limited. In this regard, it is worth mentioning that TFBSs tend to be grouped into larger regulatory sequences, the *cis*‐regulatory modules (Schmitz et al., 2022), and therefore the identification of TFBSs would benefit from the use of computational tools. Regarding *in vitro* approaches, the use of universal protein binding microarrays (uPBMs) has allowed the identification of hundreds of plant TFBSs and a clear visualization of a landscape of virtually all the plant TF families (Franco‐Zorrilla et al., 2014; Franco‐Zorrilla & Solano, 2017; Lambert et al., 2019; Weirauch et al., 2014). However, uPBMs contain synthetic oligonucleotides with pseudorandom sequences that, although very useful to define specificities, do not perfectly reproduce particular DNA composition and characteristics in different species. DNA affinity purification and sequencing (DAP‐seq) covers some shortcomings of uPBMs, as it detects genomic fragments that physically interact with the protein and it is easily scalable to different plant species (Galli et al., 2018; Gomez‐Cano et al., 2022; López‐Vidriero et al., 2021; O'Malley et al., 2016; Orduña et al., 2022). In parallel to experimental approaches, several databases collect from the literature and keep this information updated for easy access and consulting (reviewed in Kulkarni and Vandepoele, 2020), such as Cis‐BP, Cistrome, or JASPAR (Castro‐Mondragon et al., 2022; O'Malley et al., 2016; Weirauch et al., 2014).

Some bioinformatic tools, such as FIMO and RSAT's pattern matching (Bailey et al., 2009; Nguyen et al., 2018), use web‐based searches for mapping TFBSs, but they may require additional steps from the final user that are not always obvious for non‐specialists. Alternatively, other tools use pre‐computed TFBSs to analyze lists of genes and may include additional information. AthaMAP provides potential TF and small RNA binding sites in Arabidopsis (Bülow et al., 2009). PlantPan detects conserved TFBSs by pairwise comparison of two orthologous promoters (Chow et al., 2019). However, these tools require previous isolation of the gene promoters by the user, with the drawbacks indicated above. TF2Network predicts potential regulators by using available TFBSs and integrating co‐expression and protein–protein interaction data, without the need for obtaining promoters from the user (Kulkarni et al., 2018). More recently, the same group presented the Arabidopsis integrated gene regulatory network (iGRN), a learning‐based approach to infer regulatory interactions among 1491 TFs and 31 393 target genes (De Clercq et al., 2021). However, to date these methods only support Arabidopsis data. Plant Regulomics integrates transcriptomic, epigenomic, TF binding, and orthology data to exploit regulatory information from a gene list (Ran et al., 2020). Although Plant Regulomics supports information from several species, the information on plant regulatory components is still limited. PlantRegMap predicts the presence of TFBSs from a query of genes using a pre‐computed analysis in 156 species and provides information on their conservation (Tian et al., 2019). However, these analyses are restricted to orthologous TFBSs, resulting in the loss of a large number of possible TF–target interactions.

Here, we created TFBS Discovery Tool Hub (TDTHub), a quick and easy‐to‐use web‐based tool for the identification and quantification of enriched TFBSs in plants, based on a pre‐computation of TFBSs in the regulatory regions of 40 plant species, which can be easily scalable. Moreover, in contrast to other tools, TDTHub includes several options for regulatory regions and two different mapping algorithms to provide a higher versatility for the analysis of enriched TFBSs. TDTHub does not require previous isolation of promoter sequences, as it simply runs with gene IDs. These features, together with some complementary tools, make TDTHub an extremely useful tool to study transcriptional networks in plants.

## RESULTS AND DISCUSSION

### TFBS mapping in plant regulatory regions

We mapped position weight matrices (PWMs) from CisBP Build 2.00 (Weirauch et al., 2014) and from several maize (*Zea mays*) datasets (Dong et al., 2019; Galli et al., 2018; Ricci et al., 2019) in gene regulatory regions with two algorithms, Find Individual Motif Occurrences (FIMO) and Cluster‐Buster (CB). FIMO assigns a false discovery rate (FDR)‐corrected *P*‐value to motif occurrences (Grant et al., 2011), whereas CB finds dense clusters of motifs in input sequences (Frith et al., 2003). CB has been successfully applied for prediction of TF target genes in Arabidopsis (Kulkarni et al., 2018). PWM mapping was performed in 5‐kb upstream regions, starting at the translation initiation codon (TIC), as well as in introns and in 1‐kb regions downstream the translation stop codon (TSC). We favored these coordinates over the more common transcription start and end sites (TSS and TES, respectively) because those are more suitable for direct comparison among different species, especially when genome sequences are not supported by full‐length RNA sequences. It is also worth mentioning that CisBP Build 2.00 includes PWMs mostly derived from uPBM studies (Chang et al., 2013; Franco‐Zorrilla et al., 2014; Lambert et al., 2019; Weirauch et al., 2014), but also Cistrome's DAP‐seq experiments (O'Malley et al., 2016) and JASPAR 2016 (Mathelier et al., 2016) (Table S1). TFBS datasets were used to generate a plant genome‐searching database, which included information from 40 plant genomes. Although most PWMs correspond to DNA binding *in vitro*, they may reflect specificities occurring *in planta*, since binding models may intrinsically cover other specifying components outside the core motifs, as has been described for auxin response factors (ARFs) and MYC2‐related TFs (Galli et al., 2018; López‐Vidriero et al., 2021; Stigliani et al., 2019). However, the fact that TFBSs predicted *in vitro* still have some limitations with regard to the definition TF target genes cannot be overlooked.

### Evaluation of the methods and threshold setting

We considered evaluating the three variables that contribute to TFBS detection: the mapping scores, the size/type of the regulatory region, and the mapping algorithm. With regard to the first one, mappings were initially performed with score 4 for FIMO (which corresponds to FDR = 1.0E−04) and score 5 for CB, and datasets were further classified in datasets with increasing values (4, 5, and 6 for FIMO and 5, 6, and 7 for CB). Regarding the size/type of the regulatory region, we generated individual datasets corresponding to 5‐, 3‐, and 1‐kb regions upstream the TIC, with or without introns and sequences 1 kb downstream the TSC. Preliminary data using the *Solanum lycopersicum* genome as reference suggested that FIMO mappings in 5‐kb promoters or including non‐upstream sequences were useless for identifying enriched TFBSs. Although we cannot exclude the existence of regulatory sites or enhancers between −3 and −5 kb or in non‐promoter regions, the analysis of non‐proximal promoters did not significantly improve the identification of TFBSs. However, as demonstrated in previous studies (Kulkarni et al., 2018), CB performed better in regulatory regions including 5‐kb promoters, introns, and 1‐kb regions downstream the TES. These differences may be a consequence of their mapping characteristics, given that CB first identifies dense clusters of TFBSs, and these are more likely to occur in 5‐kb promoters. In fact, CB predicted dense clusters in only 6481 of 1‐kb promoters, whereas this number increased to 13 128 when considering 5‐kb promoters, supporting the relevance of detecting dense clusters prior to TFBS mapping.

**Figure 1:**
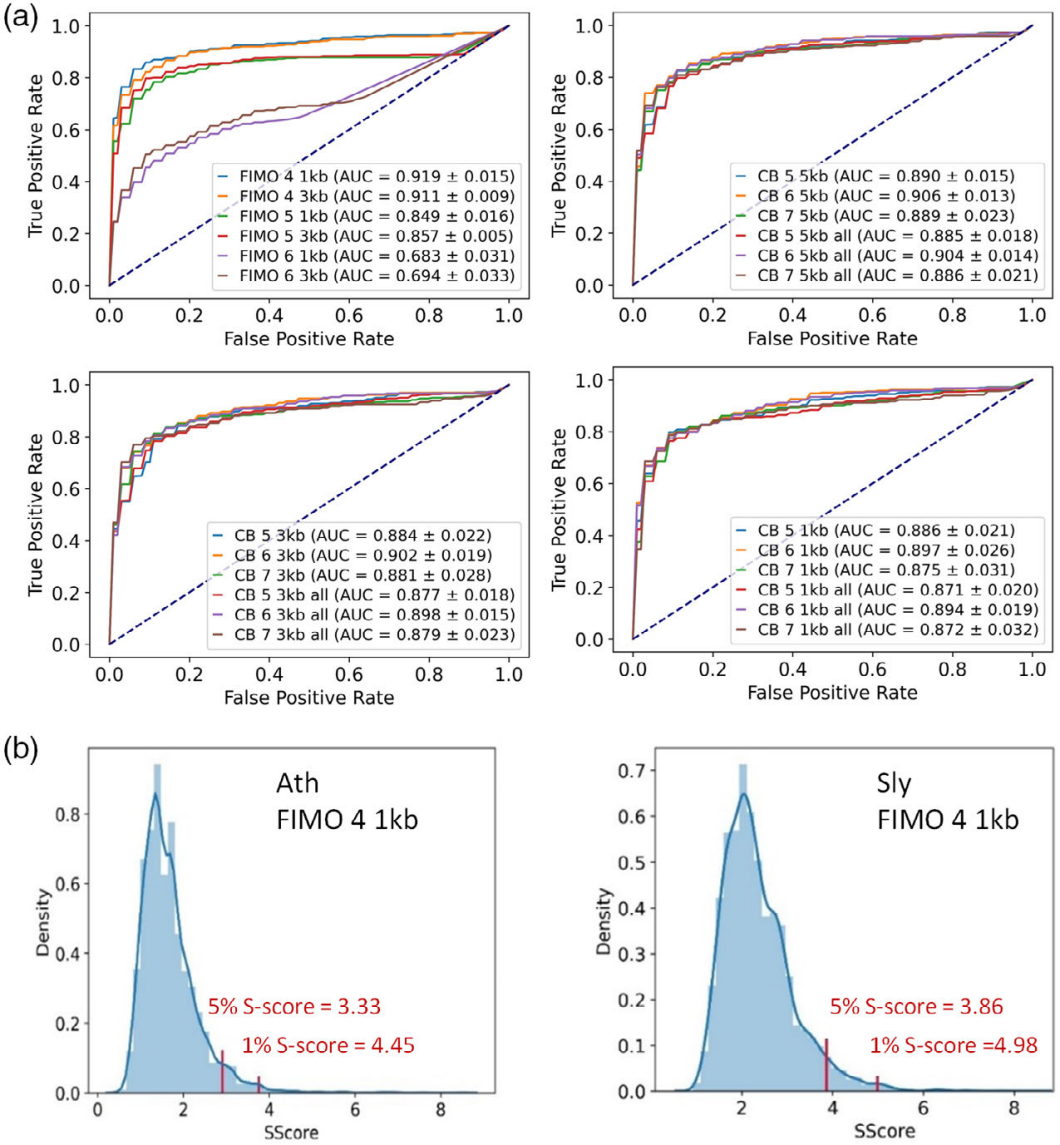
TDThub performance analysis. (a) ROC curves and AUC values for estimation of the best combinations of algorithm (FIMO and Cluster‐Buster [CB]), scores, and the upstream regulatory region size in kb. ‘All’ means that introns and downstream regions are included. (b) Density plots of the Significance Score (S‐Score) generated for estimation of the 5% and 1% threshold S‐Scores as false positive determination. Data correspond to one combination of parameters (FIMO, score 4, 1 kb upstream). Red lines represent the S‐Score values for the top 1% and 5% threshold values of the distribution that define the false thresholds. Plots correspond to S‐Score evaluation in Arabidopsis (left) and tomato (right). [Colour figure can be viewed at wileyonlinelibrary.com]

To provide a method to evaluate the power of detection of the expected TFBSs, we performed a series of analyses using 49 control sets of real TF target genes from Arabidopsis ChIP‐seq experiments (Table S2). We generated positive query lists from the TF‐bound and negative from non‐bound genes for each ChIP‐seq dataset. These lists were then used to run all possible combinations of algorithm/score and type/size of the regulatory region. We normalized the ranking position of the expected TFBS between 0 and 1, sorted by Significance Score (S‐Score), to plot receiving operating characteristic (ROC) curves and calculate their corresponding areas under the curves (AUCs). The highest AUCs corresponded to FIMO with score 4 in 1‐kb and 3‐kb (0.919 and 0.911, respectively) upstream regions (Figure 1a). Regarding CB, differences among settings were no so large, but a slight preference could be observed for 5‐kb promoters with scores 5 or 6 (0.890 and 0.906, respectively) and 3‐kb promoters with the same scores (0.884 and 0.902, respectively) (Figure 1a). The reason why higher and more restrictive scores produced lower AUCs may be due to the lower number of positive hits at high scores, with an impact on *P*‐values, and therefore on S‐Scores used for sorting and defining ranking positions. However, a balance between the number of positive hits and restrictiveness still needs to be maintained, since FIMO scanning with score 3 generated lower AUCs (Figure S1), further confirming 4 as the recommended score for initial searches.

We then evaluated whether all the combinations performed similarly when using different‐sized lists of query genes. In this case, we observed that the shortest lists (50 query genes) worked better with FIMO 4, particularly in 3‐kb promoters, followed by CB 6 in 5‐kb promoters (Figure S2). Interestingly, this trend changed with queries of 100 and 250 genes, where FIMO 4 in 1‐kb promoters (AUC 0.868 and 0.904, respectively) outperformed the AUC values of 3‐kb promoter searches (AUC 0.852 and 0.877, respectively) (Figure S2). These data agree with the fact that most of the TFBSs in Arabidopsis are located in the region between −1000 and +200 bp with respect to the TSS (Franco‐Zorrilla et al., 2014; Weirauch et al., 2014; Yu et al., 2016). By contrast, and in agreement with previous observations (Kulkarni et al., 2018), CB performed the best in 5‐kb promoters, regardless of the size of the query list.

We also aimed to evaluate the rates of false positives using the most informative combinations of algorithm and type/size of regulatory region tested. We then performed searches in 10 000 sets of randomly chosen Arabidopsis genes as input and annotated the S‐Score of the first ranking TFBS. The distributions of S‐Scores were then plotted, and we defined the threshold 5% and 1% S‐Scores as those values corresponding to the highest 5% or 1% thresholds in the distribution, respectively (Figure 1b, Figure S3). We also took into account the heterogeneity among different genomes regarding the nucleotide composition, size, or annotation quality, and we attempted to define specific threshold S‐Scores for each species. We performed the same searches in 10 000 random gene sets and defined the S‐Score thresholds at 5% and 1% for each species (Figure 1b, Figure S3, and Table S3). As expected, we observed some degree of variation among species, likely as a consequence of their heterogeneity, reinforcing the use of genome‐specific S‐Score thresholds.

Finally, we wanted to analyze whether results based on S‐Scores were comparable to those based on the simpler FDR and, in that case, which one was the more restrictive. We followed the same approach as above, but this time we applied the ranking position to TFBSs sorted by FDR to generate ROC curves. We compared two informative combinations, 3‐kb FIMO 4 and 5‐kb CB 6, and we observed that AUCs derived after sorting by S‐Score were higher (0.877 and 0.899, respectively) than those derived when the ranking corresponded to FDR (0.821 and 0.808, respectively; Figure S4), emphasizing the use of the threshold S‐Score as the preferred statistic as a false positive threshold.

Altogether, these analyses provided us with a statistical framework to select the most appropriate combinations of searching parameters in all the species contained in our database, and also contributed to increasing the confidence in TFBS searches.

### Design of TDTHub's user interface

**Figure 2:**
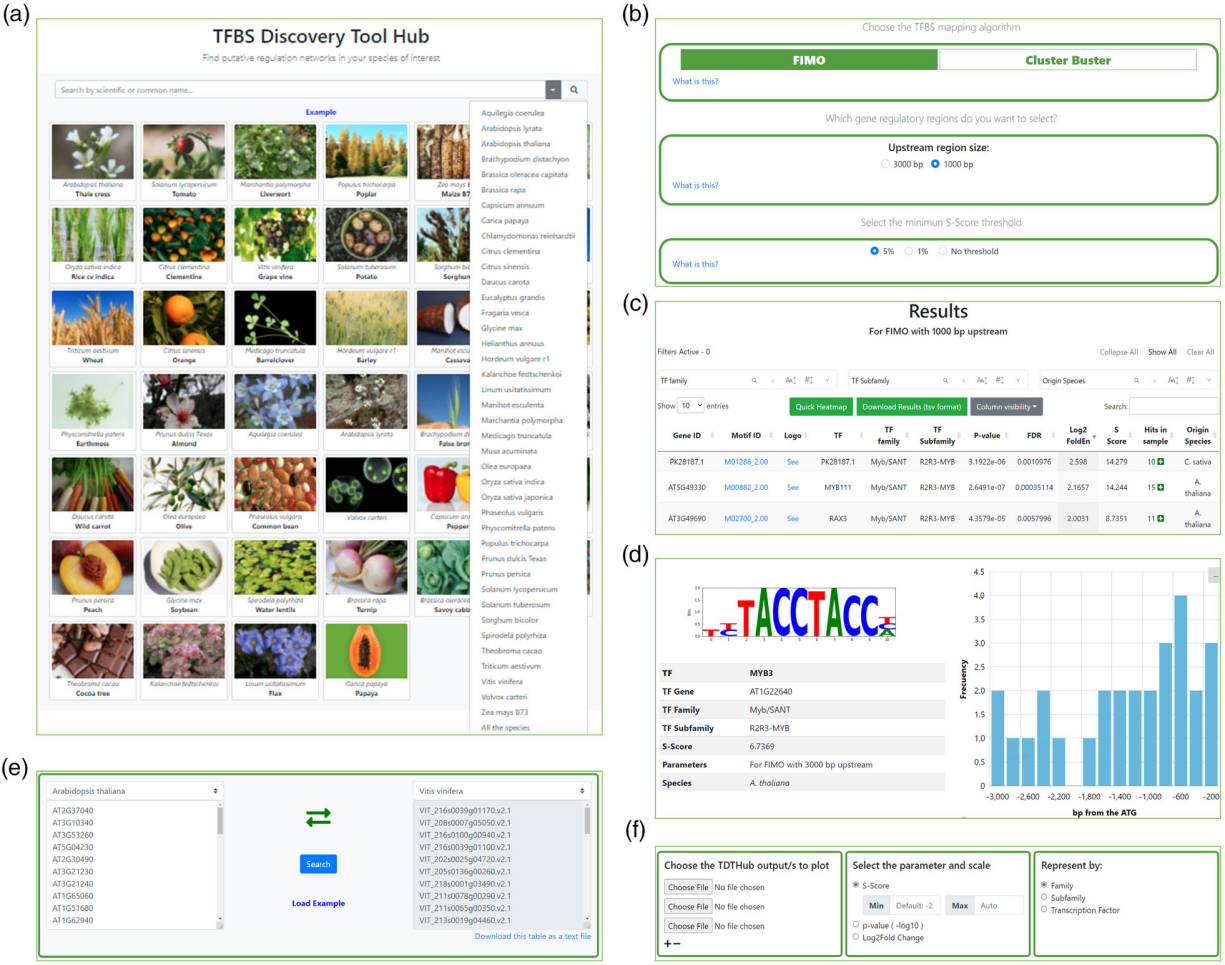
Website user interface design and functionalities. (a) Home tab with direct access to the species included in TDTHub. (b) Query tab for selection between two TFBS mapping algorithms (FIMO and Cluster‐Buster), and other parameters: upstream region length, type of regulatory regions (with or without including introns and 1‐kb downstream regions), and the S‐Score threshold. (c) Results table, with direct links to PWMs and logos. The results table can be filtered by TF family, subfamily, or species, and the Results table can be customized by showing/hiding selected columns. The symbol ‘+’ opens the list of positive hits for each motif and a new menu for coordinate searching. Default sorting is by S‐Score, but any field may be used for sorting. (d) TFBS card with the complete information and a histogram showing the distribution of positive hits in promoters. (e) BLASTP tool tab for searching the most similar genes by reciprocal BRH between Arabidopsis and any other species, and *vice versa*. (f) Interactive Heatmap tab. The left menu allows loading TDTHub results tables from different searches (up to 20). The middle menu selects scale preferences and the type of parameter to plot (*P*‐value, fold enrichment, or S‐Score). The right menu shows TF representation preferences by family, by subfamily, or individually. [Colour figure can be viewed at wileyonlinelibrary.com]

Our goal was to create a web‐based tool for quick and intuitive access for non‐experienced researchers interested in the transcriptional control of sets of co‐expressed or co‐regulated genes, or of genes that belong to the same metabolic or developmental pathway. Therefore, we generated an interface where the final user may choose among several options: (i) species of interest; (ii) mapping algorithm (FIMO or CB); and (iii) size and type of the regulatory regions (Figure 2a,b). With these options, we pursued generating a balanced tool between flexibility and ease of use, in order to maximize the amount of information provided. The results table includes the TFBS information (motif ID, TF, family/subfamily, and origin species), the statistical values associated with enrichment (fold enrichment over the expected value by chance, *P*‐value, FDR, and S‐Score), and the number of positive hits (Figure 2c). The table is interactive, allows for searching and sorting by any field, and links directly to the PWMs and logos for each motif. An additional feature of interest is the possibility to retrieve the lists of genes containing the TFBS of interest, their coordinates relative to the TIC, and a histogram with a graphical representation of TFBS locations along the promoter (Figure 2c,d).

Along with the main tool, we included two additional tools to help analyze and visualize regulatory characteristics of query genes. One of the tools performs rapid searches for highly homologous genes between Arabidopsis and the rest of the species, and *vice versa*. The method for assigning gene homology is based on best reciprocal BLASTP hit (BRH) which, although it does not consider collinearity across plant genomes, has been widely used in the identification of orthologs (Tatusov et al., 1997; Tian et al., 2019). This tool is accessible from the navigation bar in the home tab menu, and BRH genes can be directly launched to the TDTHub tool for enrichment analysis (Figure 2d). We also provide an interactive heatmap tool to represent fold enrichment values of TFBSs, *P*‐values, or the S‐Score, with customizable color scales. This tool is fully compatible with the table generated within the TDTHub main search tab and supports up to 20 results tables (Figure 2e). TFBSs may also be classified into families/subfamilies (mean values represented) to simplify visualization of groups of enriched TFBSs.

### TDTHub benchmark

**Figure 3:**
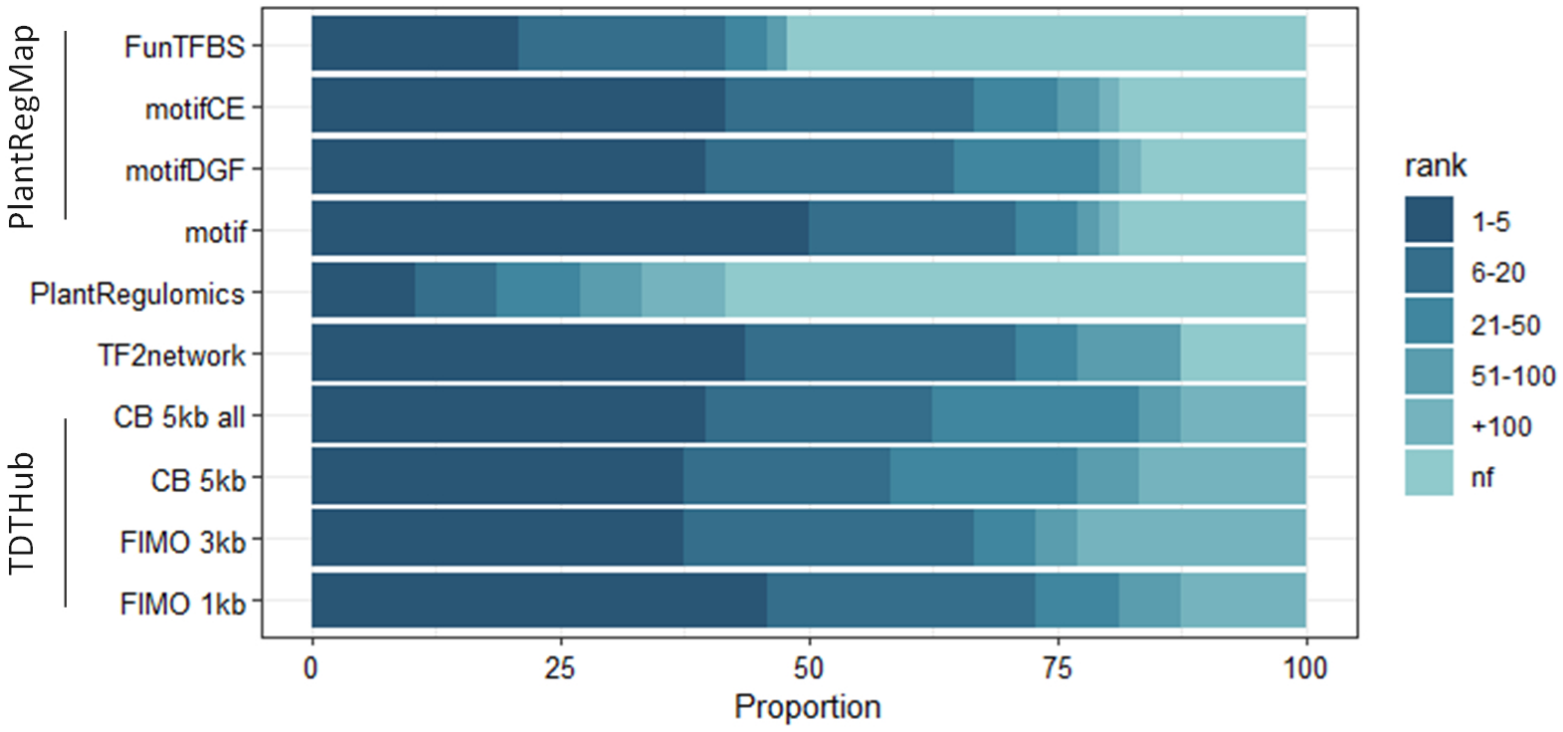
TDTHub performance in comparison with different tools. Bars represent the proportions of the expected TFs recovered at different ranking positions. TDTHub was applied with the four most informative settings: FIMO 1 kb, and FIMO 3 kb, and CB 5 kb with (all) or without introns and 1‐kb downstream regions. PlantRegMap was used in the four settings available (Tian et al., 2019): motif, FIMO searches in the 0.5‐kb region upstream of the TSS; motifDGF, motif overlapping with DNase I footprints; motifCE, motif overlapping with conserved elements; FunTFBS, functional TFBS. [Colour figure can be viewed at wileyonlinelibrary.com]

We were interested in comparing how well the TDTHub tool performed for the identification of enriched TFBSs in comparison to other available tools. In these tests, we chose TF2Network, since it is one of the simplest and most effective methods of use for TFBS enrichment in Arabidopsis (Kulkarni et al., 2018). We also included Plant Regulomics, which carries the additional advantage of being able to perform analysis in several species (Ran et al., 2020). We also evaluated PlantRegMap, a portal that includes several tools for gene regulation analysis in plants (Tian et al., 2019). For homogeneous comparison of the tools, we used a number of control datasets from published Arabidopsis ChIP‐seq experiments (Table S4), and searched for enriched TFBSs with four options in TDTHub and PlantRegMap, as well as with TF2Network and Plant Regulomics. Our results showed that TDTHub performed similarly to TF2Network and PlantRegMap for the identification of the TFs involved in the regulation of control datasets, particularly using FIMO with 1‐kb promoters. This effect was observed for ranking positions between #1 and #5, and especially up to #20 (Figure 3). Moreover, TDTHub performed better than Plant Regulomics in the identification of enriched TFBSs, in which the detected motifs were similarly distributed across all the rank intervals (Figure 3).

Therefore, on the one hand, TDTHub performed similarly to TF2Network, with the difference that the latter only supports Arabidopsis data and one fixed searching parameter. By contrast, TDTHub represents 40 species and has a greater versatility for TFBS searching, as it allows two different parameters with two sizes/types of regulatory regions. On the other hand, PlantRegMap tools are all based on FIMO mappings in 500‐bp regions upstream the TSS as the fixed parameter, and PlantRegMap supports analysis of up to 156 plant species. However, enrichments analysis in PlantRegMap is performed on a pre‐computed list of conserved TFBSs that represents only a small fraction of the total motifs in a plant genome. The versatility offered by TDTHub would be of interest given the casuistry in gene regulation and the fact that TFBSs may not be similarly distributed in all the target genes and in all the species. In our data, FIMO performed better in 1‐kb promoters than in 3‐kb promoters (Figure 3), but in some datasets this was the opposite, such as in ABF3 and ATHB7, where the expected TFBS appeared in positions #25 and #9, respectively, in 1‐kb promoter regions and lowered down to #8 and #3, respectively, when 3‐kb promoter regions were analyzed.

### Case study 1: transcriptional regulation of genes in the anthocyanin biosynthesis pathway

To illustrate the functionalities in TDTHub, we focused on the anthocyanin biosynthesis pathway, as it is one of the best‐studied pathways in plants (Vogt, 2010; Xu et al., 2015). Starting from phenylalanine, several steps are shared by three major branches leading to the production of lignins, lignans, and flavonoids, the latter including anthocyanins (Vogt, 2010). Transcription of the enzyme‐coding genes is controlled by two types of regulators, ternary MBW (for MYB‐R2R3–basic helix–loop–helix [bHLH]–WD40) complexes and individual MYB‐R2R3 factors (Xu et al., 2015). We identified the genes involved in the flavonoid and anthocyanin pathways in Arabidopsis from the Kyoto Encyclopedia of Genes and Genomes (KEGG) database (pathways map00941 and map00942, respectively) (Kanehisa et al., 2017) and used them for TFBS enrichment analysis (Table S5). FIMO searches in 1‐kb promoters revealed the prevalence of MYB‐R2R3 TFs, given that 92% and 84% of the top motifs from the anthocyanin and flavonoid gene lists, respectively, belonged to this TF subfamily (Tables S6 and S7).

**Figure 4:**
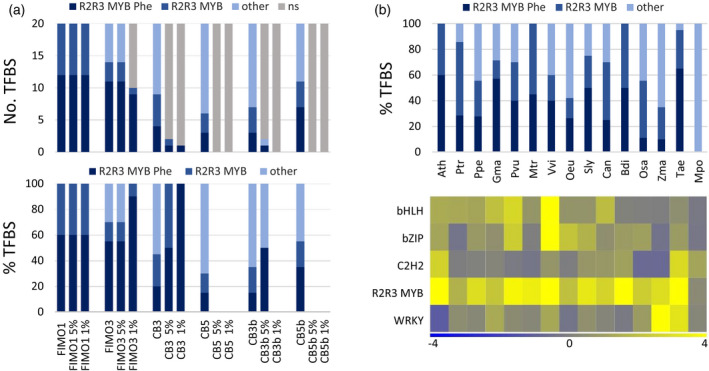
Involvement of R2R3‐MYBs in the phenylpropanoid and anthocyanin pathways. (a) Number (top) and proportion (bottom) of TFBSs corresponding to R2R3‐MYBs among the top 20 motifs, sorted by S‐Score. R2R3‐MYBs involved in the anthocyanin pathway are shown in dark blue, and those implicated in the flavonoid pathway are shown in intermediate blue. The lightest blue corresponds to TFBSs other than R2R3‐MYBs, whereas TFBSs with S‐Score greater than the 5% threshold are shown in gray (ns). Proportions are calculated after removing ‘ns’ motifs. Each group of bars corresponds to a different searching combination: FIMO1/CB1, FIMO3/CB3, and CB5 used 1‐, 3‐, and 5‐kb upstream regions, respectively. CB3b/CB5b included upstream, intron, and 1‐kb downstream regions. (b) (Top) Proportion of TFBSs corresponding to R2R3‐MYB proteins among the top 25 PWMs in the searches with orthologs using FIMO 1 kb upstream and filtered by S‐Score threshold < 5%. Ath, *Arabidopsis thaliana*; Ptr, *Populus trichocarpa*; Ppe, *Prunus persica*; Gma, *Glycine max*; Pvu, *Phaseolus vulgaris*; Mtr, *Medicago truncatula*, Vvi, *Vitis vinifera*; Oeu, *Olea europaea*; Sly, *Solanum lycopersicum*; Can, *Capsicum annuum*; Bdi, *Brachypodium distachyon*; Osa, *Oryza sativa* var *japonica*; Zma, *Zea mays*; Tae, *Triticum aestivum*; Mpo, *Marchantia polymorpha*. (Bottom) Heatmaps of enriched TFBSs, clustered by subfamilies, of the anthocyanin pathway genes from all the species. Heatmaps represent the average S‐Scores for the 25 top‐ranking TFBSs from each species. TF families represented by just one TFBS in the query lists were removed for better visualization. [Colour figure can be viewed at wileyonlinelibrary.com]

We then focused on the 38 genes from the anthocyanin branch to further evaluate the performance of different searching parameters. We observed a high number of MYB‐R2R3 motifs, particularly of those involved in the phenylpropanoid pathway (Figure 4a), such as the maize P gene (Grotewold et al., 1994), or MYB55, MYB99, and MYB111, which control different branches of the synthesis pathway of flavonoids (Battat et al., 2019; Kishi‐Kaboshi et al., 2018; Stracke et al., 2007). These data indicate that TDTHub is able to detect subtle differences between different TFBSs from the same family/subfamily, reinforcing the power of bioinformatic tools for the identification of specific TFs. We obtained higher proportions of MYB‐R2R3 motifs in FIMO and, importantly, after filtering for threshold S‐Score < 5% or 1% (Figure 4a). S‐Score corrections in CB searches drastically reduced the number of significant motifs (Figure 4a), most likely due to the low number of query genes and the searching methodology of this algorithm.

By using the reciprocal BLASTP tool, we obtained the likely orthologs in 14 species, including dicots, monocots, and the liverwort *Marchantia polymorpha* (Marchantia), and used these genes for TFBS enrichment analysis with FIMO (1 kb upstream; S‐Score < 5%). With the exception of *Marchantia*, we obtained 35 to 100% MYB‐R2R3 TFBSs among the top motifs and from these, 38% on average corresponded to flavonoid‐related MYBs (Figure 4b). We selected the top‐ranking motifs from each species and generated a final list to plot heatmaps with the visualization tool provided, and confirmed that the R2R3‐MYB subfamily showed the highest S‐Score (Figure 4b). This observation agrees with the conservation of the regulation of the phenylpropanoid pathway, but only a few R2R3‐MYB TFBSs are marginally enriched in the *Marchantia* anthocyanin input set (Figure 4b). This could be due to the low number of BLASTP orthologs (19 out of 38 genes) and to the fact that promoters may be longer in this species compared to those of higher plants (López‐Vidriero et al., 2021). Alternatively, there may be some differences in DNA binding specificity between vascular and non‐vascular plant TFs. In fact, the only TF known to act as a key regulator of flavonoid production in *Marchantia*, MpMYB14, differs from its vascular orthologs in key residues involved in MYB–bHLH interaction that could affect DNA binding (Albert et al., 2018).

### Case study 2: transcriptional regulation of the jasmonate signaling pathway

**Figure 5:**
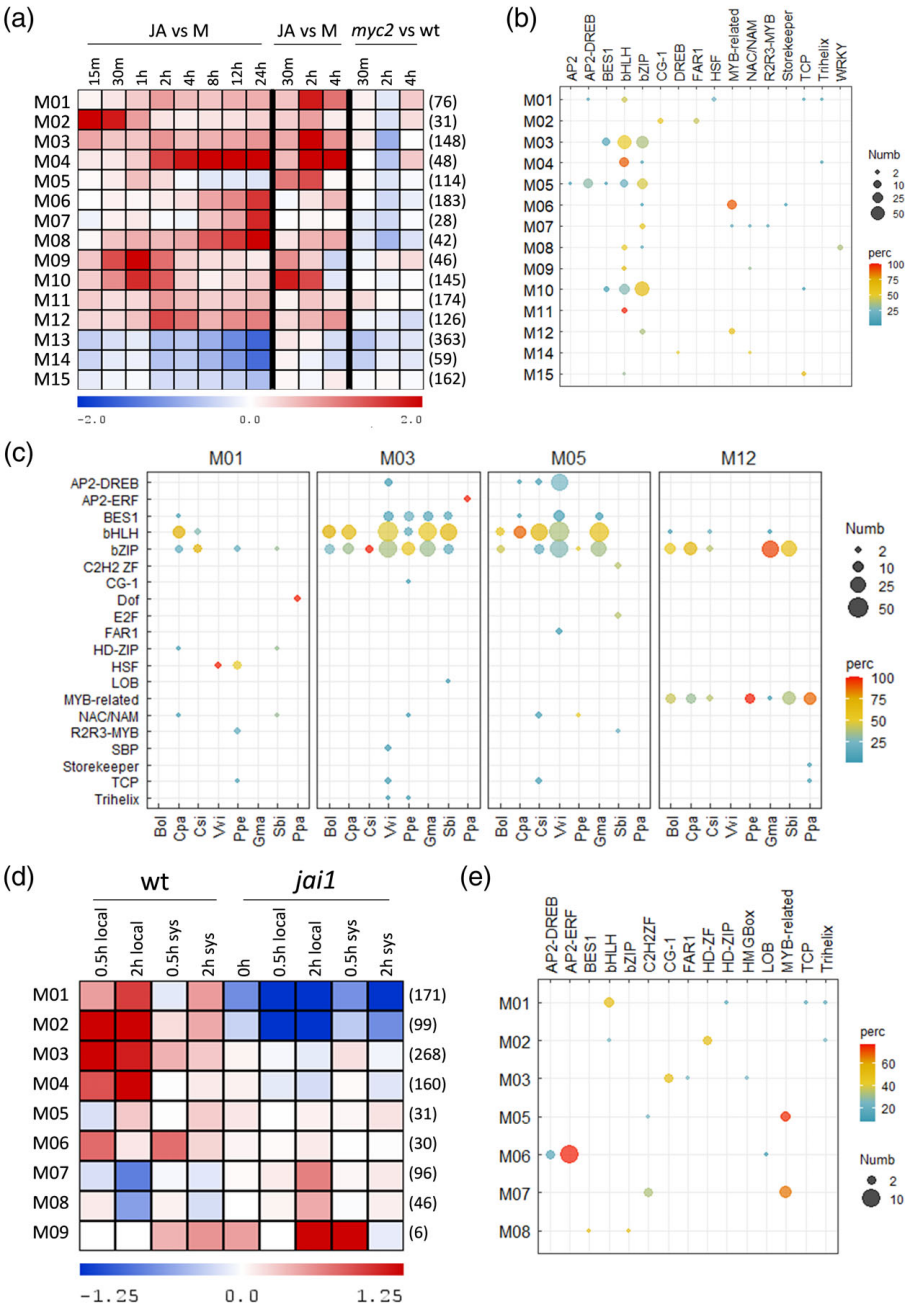
TDTHub‐based study of transcriptional regulation in response to jasmonates (JAs). (a) Heatmap summarizing the expression of the modules (names on the left) obtained after WGCNA clustering in Arabidopsis. The number of genes in each module is indicated between brackets on the right. Samples represent gene expression comparisons of plants treated with JA during the period indicated in hours (h) versus the mock treatment (in log_2_ scale). The last three columns correspond to the comparison of the *myc2* mutant vs. wild‐type plants at different time points. (b) Number (Numb) and percentage (perc) of TFBSs belonging to each of the families indicated (top) that were significantly enriched in each module in (a). Searches were performed with FIMO 1 kb and TFBS selected at S‐Score threshold < 1%. (c) Number and percentage of TFBSs from each family (left) in the lists of orthologs for the modules indicated (top) from several species (bottom). TFBSs were filtered based on the corresponding S‐Score threshold < 1% for each species. Bol, *Brassica oleracea*; Cpa, *Carica papaya*; Vvi, *Vitis vinifera*; Ppe, *Prunus persica*; Gma, *Glycine max*; Stu, *Solanum tuberosum*; Sbi, *Sorghum bicolor*; Ppa, *Physcomitrella patens*. (d) Heatmap summarizing the expression of the modules obtained after WGCNA clustering in tomato. Samples correspond to the log_2_(fold change) of treatment vs. mock (wt) or *jai1* vs. wt (*jai1*) at the indicated time points. The number of genes from each module is shown between brackets on the right. (e) Number and percentage of TFBSs belonging to each of the families indicated (top) that were significantly enriched in the modules from (d). Searches were performed with FIMO, score 4, 1 kb upstream, and TFBSs selected at S‐Score threshold <1%. [Colour figure can be viewed at wileyonlinelibrary.com]

We evaluated TDTHub in the well‐known transcriptional pathwayunderlying jasmonate (JA) signaling, with the aim of identifying novel TFBSs and their putatively TFs involved. We used available transcriptomic data from a time series in response to JA and from the loss‐of‐function mutant *myc2* (Zander et al., 2020), and performed a weighted correlation network analysis (WGCNA) for finding modules of highly correlated genes (Table S8). Then, we used TDTHub to search for enriched TFBSs in each of the modules (FIMO 1 kb). We observed large enrichments in bHLH motifs in the modules corresponding to genes induced shortly after JA treatment and that depend on MYC2 activity (Figure 5a,b). These data agree with the main role of MYC2 (and related) bHLH proteins in transcriptional activation, through recognition of the G‐box, during the initial steps after hormone perception (Chini et al., 2007; Fernández‐Calvo et al., 2011; Godoy et al., 2011; Hickman et al., 2017; Wang et al., 2019; Zander et al., 2020). It is worth noting the enrichment of bZIP and BES1 motifs in these modules (Figure 4a,b), but it must be considered that these may result, at least partially, from the similarity of these motifs to the G‐box. However, the higher enrichment of bZIP motifs in the modules M10 and M12 suggests a role of bZIP factors, supported by the presence of the bZIPs At1g19490 (bZIP62) and HY5 in M10 and HYH and ABF1 in M12 (Table S8), as was demonstrated for HY5 (Ortigosa et al., 2020). Enrichment of AP2 DREB‐type motifs in M05 coincides with Rap2.7 and ERF53 in this module, although we cannot discard the possibility of the regulation of these genes by ORA47 (Pauwels et al., 2008; van Moerkercke et al., 2019), whose module M02 immediately precedes in expression to M05 (Figure 5a). Similarly, MYB‐related elements are enriched in M12, which agrees with REVEILLE4/LHY–CCA1–Like1 (RVE4/LCL1) in this module (Figure 5b; Table S8), supporting a link between JA‐derived responses and the circadian rhythm (Zhang et al., 2018; Zhang et al., 2019). Finally, the enrichment of HSF motifs in M01 is remarkable, and the presence of HSFA2, HSFB2A, and HSFB2B factors in this module is in agreement with the recently described role of JAs in plant thermotolerance (Monte et al., 2020; Zhu et al., 2021).

We used the BLASTP tool to obtain the likely orthologs in different species and conducted new TFBS analyses for some modules (FIMO 1 kb). We observed a parallelism of TFBS enrichment between Arabidopsis and other species, particularly dicots, but also in monocots and mosses (Figure 5c). The parallelism was more evident in bHLH (or BES1 and bZIP) motifs, in agreement with the conserved role of MYCs (Du et al., 2017; Kazan & Manners, 2013; López‐Vidriero et al., 2021; Peñuelas et al., 2019). Of particular interest, the MYB‐related function was significantly prevalent in the regulation of the module M12 (Figure 5c) and HSFs are involved in M01. The lack of enrichment in some species is likely a result of the reduced number of queried genes after searching for orthologs, especially in modules with a lower number of genes.

To further evaluate TDTHub, we performed an additional WGCNA and TFBS enrichment study in tomato. The transcriptomic assay consisted of a time series in response to wounding in wild‐type plants and in the *jai1* mutant, which lacks the tomato JA co‐receptor CORONATINE‐INSENSITIVE1 (COI1) (Katsir et al., 2008; Li et al., 2004). DNA motifs for bHLHs were highly enriched in modules M01 and M02, which correspond to genes with the highest expression in wounded leaves and the lowest in the *jai1* mutant (Figure 5d,e). Consistently, the genes Solyc01g096370 in module M01 and Solyc01g096050 and Solyc10g009290 in module M02 are MYC2‐related genes, supporting their main role in the response to wounding in this species (Du et al., 2017; López‐Vidriero et al., 2021) (Table S9). Furthermore, the large enrichment of AP2‐ERF motifs in M06 agrees with the presence in this module of the tomato ETHYLENE‐RESPONSIVE TRANSCRIPTION FACTOR 1 (ERF1, Solyc05g050790), supporting the participation of a JA/ethylene‐dependent pathway in the regulation of these genes (Figure 5e, Table S9) (Lorenzo et al., 2003). Besides, some other TF–TFBS pairs may be operating in these conditions. For example, the zinc‐finger homeodomain (ZF‐HD) gene Solyc09g089550 might regulate the expression of M02 genes. On the other side, the MYB‐related gene Solyc01g109690 in M07 may have a role in downregulating expression in response to wounding of COI1‐dependent genes (Figure 5d,e; Table S9). This gene corresponds to RADIALIS‐LIKE 6, a small protein containing a MYB‐like DNA binding domain whose function has not been studied in depth. The similar *RADIALIS‐LIKE SANT/MYB 1* (*RSM1*) in Arabidopsis has been implicated in the regulation of growth under red light and in maintaining auxin signaling and homeostasis (Hamaguchi et al., 2008; Yang et al., 2018), several aspects in which MYC2 and related proteins have been implicated (Ortigosa et al., 2020). It would be interesting to study in the future the role of the Solyc01g109690 ortholog in Arabidopsis, in particular its putative role in mediating light‐dependent growth and defense mechanisms against stress and pathogens.

In summary, through analysis of Arabidopsis and tomato, TDTHub has been shown to be very efficient in determining the most relevant TFBSs and putative TFs involved in the regulation of groups of genes, strengthening our knowledge of the participation of some well‐known TFs, and in discovering new transcriptional cascades involved in the regulation of the responses to JA or wounding. These analyses have been performed using stringent criteria (a threshold S‐Score of <1%) and with just one combination of search parameters (FIMO 1 kb). Although we cannot exclude the existence of additional DNA motifs enriched, evidenced for example after filtering with less stringent criteria, using longer promoters or the CB method, these examples illustrate the utilities of TDTHub in the study of transcriptional pathways. Furthermore, TDTHub provides a rapid alternative for an initial assessment of *cis*‐motif conservation between Arabidopsis and other species by using the BLASTP tool followed by TFBS searches, which could be of the interest in studies on the involvement of TFs during speciation and/or plant domestication.

## CONCLUDING REMARKS

TDTHub is a web tool for rapid identification of enriched TFBSs and putative TFs involved in the regulation of genes of interest and has several features that justify its use in transcriptional regulatory studies in plants, especially for non‐experienced users. These include: (i) pre‐processed data for 40 relevant plant species are included, and the number of species will increase in future planned updates; (ii) two mapping algorithms with different sizes and types of regulatory regions are implemented, providing greater versatility to account for the varied casuistry on TFBS architecture in different species; and (iii) additional useful tools are available, such as a rapid search tool for likely orthologs and interactive heatmaps for clear visualizations of TFBS results and coordinates of enriched TFBSs in gene promoters. Therefore, the main features of TDTHub (ease of use, quickness, and versatile analysis in different species) make it an extremely useful tool for the analysis of transcriptional pathways in plants, independent of the bioinformatic skills of the researcher. TDTHub is accessible at http://acrab.cnb.csic.es/TDTHub/.

## EXPERIMENTAL PROCEDURES

### Collection of TFBSs, genomes, and regulatory regions

We mapped 1564 PWMs representing TFBSs from direct experiments involving TFs from 52 different photosynthetic eukaryotic species, obtained from the CisBP database (updated on January 8, 2019; database build 2.00) (Weirauch et al., 2014). This version includes PWMs mainly derived from PBMs (Franco‐Zorrilla et al., 2014; Weirauch et al., 2014), but also from JASPAR (Mathelier et al., 2016) and Cistrome's DAP‐seq data (O'Malley et al., 2016). In total, 117 maize PWMs derived from ChIP‐seq were taken from the original work (Dong et al., 2019), whereas PWMs from DAP‐seq experiments were generated using MEME with the command ‘‐dna ‐mod zoops ‐nmotifs 1 ‐bfile ‐revcomp ‐minw 8 ‐maxw 16’ and a background of Markov model 0 calculated from the *Z. mays* B73.4.46 genome.

Genome fasta sequences and General Feature Format (GFF) annotations were obtained from different databases (Table S10). We used Python3 and BEDTOOLS version 2.23 (Quinlan, 2014) to extract desired gene regions and to build the inputs for prediction tools, according to the following design. (i) A gene regulatory structure includes 5000 bp upstream of the TIC and 1000 bp downstream of the TSC, as well as introns. (ii) If a neighboring gene overlaps a basic regulatory structure, this sequence is trimmed out to extract purely intergenic fragments. (iii) In case of overlapping genes in different strands, standard 5000 bb upstream and 1000 bp downstream are considered. (iv) The largest splicing variant of each gene is considered as representative gene model. (v) Introns larger than 5000 bp in length are skipped to avoid annotation mistakes and unreasonably long computing times. Translation initiation and stop codons were selected as reference points for more consistency of gene sequences between species and as some genes may not contain 5′‐untranslated regions.

### PWM mapping using FIMO and Cluster‐Buster

The algorithm FIMO (Grant et al., 2011) was used with default parameters except for the ‘‐‐max‐strand’ option (MEME version 5.10.0). In CB (Frith et al., 2003), the −m (motif score threshold) parameter was set to 5, and ‐p (pseudo counts) was set to 0. To ensure higher versatility, the analysis were performed with three different gene regions for CB: 5000 bp upstream, introns, and 1000 bp downstream.

### Statistical analysis

Motif enrichment was evaluated with the *P*‐value calculated applying the hypergeometric distribution, using the probability mass function as follows:$P\left(x,M,n,N\right)=\frac{\left(\begin{array}{c} n\\ k \end{array}\right)\left(\begin{array}{c} M-n\\ N-k \end{array}\right)}{\left(\begin{array}{c} M\\ N \end{array}\right)},$ where*n* = the number of genes in the input set, *M* = the number of genes in the whole genome, *N* = the number of genes with a match for a specific PWM in the whole genome,and x = the number of genes in the input set with the PWM match.

The FDR of enrichment was determined using Benjamini–Hochberg correction (Benjamini & Hochberg, 1995). Fold enrichment (FE) was calculated for each motif as a base 2 logarithm using the following function:

$\mathrm{FE}=\log_{2}\frac{\left(\frac{x}{N}\right)}{\left(\frac{n}{M}\right)}.$

The S‐Score (Gearing et al., 2019) was defined as follows:

$\text{Significance Score}=-\log_{10}{\left(P-\text{value}\right)}^{*}\mathrm{FE}.$

### ChIP‐seq and RNA‐seq datasets analyzed in this study

In total, 49 ChIP‐seq datasets taken from the literature are summarized in Table S2. We defined as targets the genes containing at least one significant peak in the interval between 3000 bp upstream the TSS to 1000 bp downstream the TES, including the gene body. Significant peaks were annotated with genomic coordinates relative to the TSS with ChIP Seeker (Yu et al., 2015). ChIP‐seq datasets for TDTHub benchmarking were selected if the TF involved was included in our database and the adjusted *P*‐value information was included in the publication. Final lists corresponded to the top 500 targets, sorted by MACS2 adjusted *q*‐value. Control datasets from (Kulkarni et al., 2018) that met the same criteria were also included in our benchmark.

The RNA‐seq dataset from Arabidopsis was previously published (Zander et al., 2020) and raw reads were obtained from GEO (GSE132316). Reads were cleaned with Trim Galore and mapped to the reference genome with HISAT2 (Kim et al., 2015). Differential expression was assessed with EdgeR (Robinson et al., 2010) and significant genes were selected with log_2_(fold change) > |1| and FDR < 0.01 in at least one of the comparisons. The RNA‐seq dataset from tomato (Liu et al., 2018) was analyzed as before and genes with log_2_(fold change) > |1.5| and FDR < 0.01 were selected. Normalized counts of differentially expressed genes were used as input for WGCNA and genes in modules were filtered and relocated for module membership > 0.8 (Langfelder & Horvath, 2008).

### Benchmark datasets

To compare the performance of different combinations of parameters, input list sizes, and sorting methods, we generated a positive query list for each of the 49 ChIP‐seq experiments (Table S2) and a negative query list for the non‐bound genes for each ChIP‐seq experiment. Each query list consisted of 50, 100, 250, or 500 genes randomly chosen from their corresponding gene pool to correct for large ChIP‐seq gene target lists. We also made 10 replicates to reduce possible bias caused by gene subsampling. Then, we ran FIMO and CB with several combinations of scores and regulatory regions, and these tables were sorted by S‐Score or FDR. Finally, we plotted in a ROC curve the ranking position threshold, scored between 0 and 1, of the expected or more similar motif for all the pairs of positive and negative labeled query lists.

To empirically estimate a false positive S‐Score threshold, we ran the most informative combinations of algorithm and promoter type and size, using as query 10 000 background lists of 500 randomly chosen genes. We annotated the S‐Score of the top scoring TFBSs to obtain the distribution of false positive S‐Scores, from which we defined the 5% and 1% threshold values. This analysis was performed in the 40 species to obtain the list of threshold S‐Scores for each species.

### Identification of BRH genes

Likely orthologs or BRH genes between Arabidopsis and the rest of the species were obtained using the best BRH method. We first obtained all the protein sequences for each genome by translating the gene sequences from GFFs. BLASTP (Altschul et al., 1990) was run for pairs of ‘proteomes’ using parameters ‐evalue 1e‐10, −max_hsps 1, −max_target_seqs 5, and reciprocal matches were filtered.

### Design and programming of the web tool interface and database

The web tool interface was developed using Bootstrap (Version 4.5.0; https://getbootstrap.com/). The data is stored in a PostgresSQL database and Django (version 3.1; https://www.djangoproject.com/) was chosen to develop the website, hosted in an Apache server. Results tables were created using DataTable (https://datatables.net/manual/index) and the Heatmap tool was created using Amcharts (version 4; https://www.amcharts.com/docs/v4/tutorials/multi‐color‐xy‐heatmap/) and JavaScript. The website has been tested in the current last stable versions of Chrome, Firefox, Safari, and Microsoft Edge.

## AUTHOR CONTRIBUTIONS

JMFZ conceived the research. JG and JMFZ performed the research, analyzed data, and designed the website. JG wrote the code. JMFZ wrote the manuscript with input from JG.

## CONFLICTS OF INTEREST

The authors declare that they have no conflicts of interest associated with this work.

## Supporting information


**Figure S1.** Estimation of all the combinations’ FIMO scores and promoter sizes.
**Figure S2.** Estimation of the best combinations of algorithm, scores, and the upstream regulatory region size.
**Figure S3.** Estimation of the 5% and 1% threshold S‐Scores as false positive evaluation.
**Figure S4.** Estimation of the best filtering values, S‐Score, and FDR.

**Table S1.** PWMs used in this study and related information.
**Table S2.** ChIP‐seq datasets used in this work for benchmarking.
**Table S3.** Threshold S‐Scores at 5% and 1% for each species.
**Table S4.** Lists of 500 targets from published ChIP‐seq experiments used for comparison of tools.
**Table S5.** Arabidopsis genes coding for enzymes involved in the flavonoid pathway.
**Table S6.** Top 50 TFBSs using as query the subset of genes implicated in the anthocyanin biosynthesis pathway.
**Table S7.** Top 50 TFBSs using as query the subset of genes implicated in the phenylpropanoid biosynthesis pathway.
**Table S8.** WGCNA modules in the transcriptomic assay in response to jasmonate (JA) in Arabidopsis.
**Table S9.** WGCNA modules in the transcriptomic assay in response to wounding in wild‐type and *jai1* mutant tomato.
**Table S10.** Source of FASTA and GFF files and genome versions of the species included in TDTHub.

## Data Availability

All relevant data can be found within the article or the supporting information and on the TDTHub web page (http://acrab.cnb.csic.es/TDTHub/).
