# Supplementary material for: TDTHub, a web server tool for the analysis of transcription factor binding sites in plants
Source: Plant J. 2022 Jul 1;111(4):1203–15. doi: 10.1111/tpj.15873 (PMC9541588; doi:10.1111/tpj.15873)
Supplement: Supplementary file 1 — Figure S1. Estimation of all the combinations’ FIMO scores and promoter sizes. Figure S2. Estimation of the best combinations of algorithm, scores, and the upstream regulatory region size. Figure S3. Estimation of the 5% and 1% threshold S‐Scores as false positive evaluation. Figure S4. Estimation of the best filtering values, S‐Score, and FDR. [file TPJ-111-1203-s001.pdf]

Figure S1

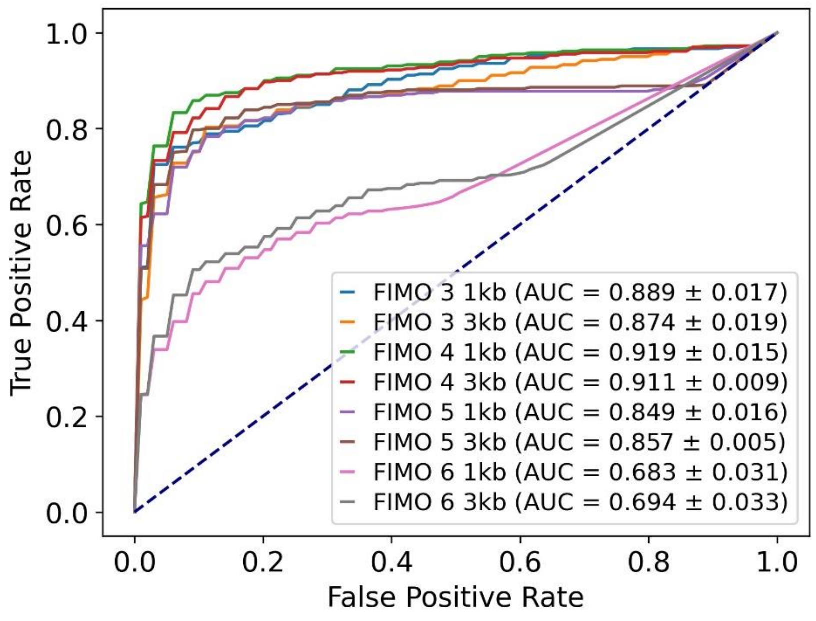

Figure S1. ROC curves and AUC values for estimation of all the combinations FIMO scores, including 3, and promoter sizes.

Figure S2

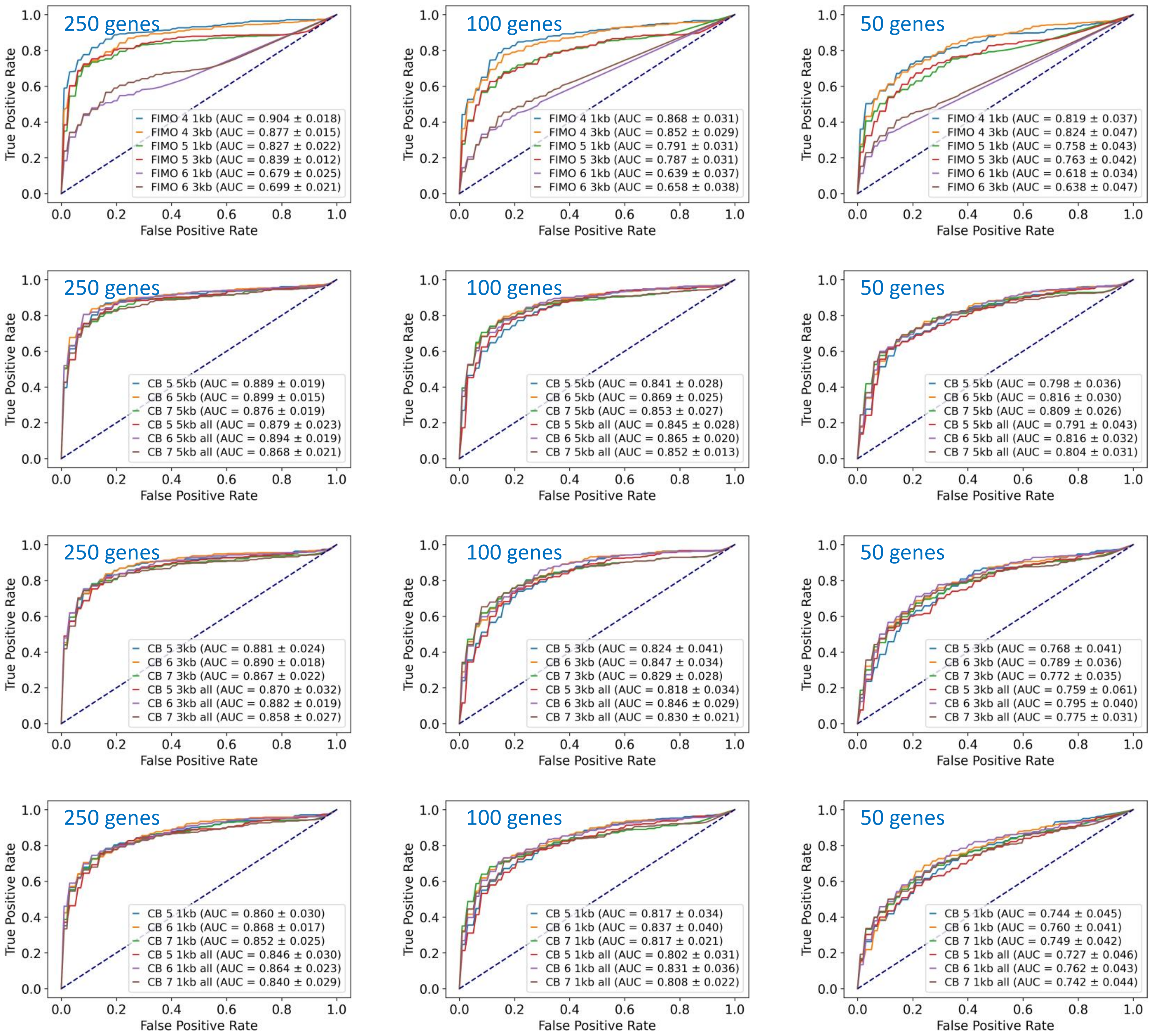

Figure S2. ROC curves and AUC values for estimation of the best combinations of algorithm (FIMO and Cluster Buster, CB), scores and the upstream regulatory region size in kb. 'All' means that introns and downstream regions are included. Searches were performed with lists of 50, 100 and 250 genes from control datasets.

Figure S3

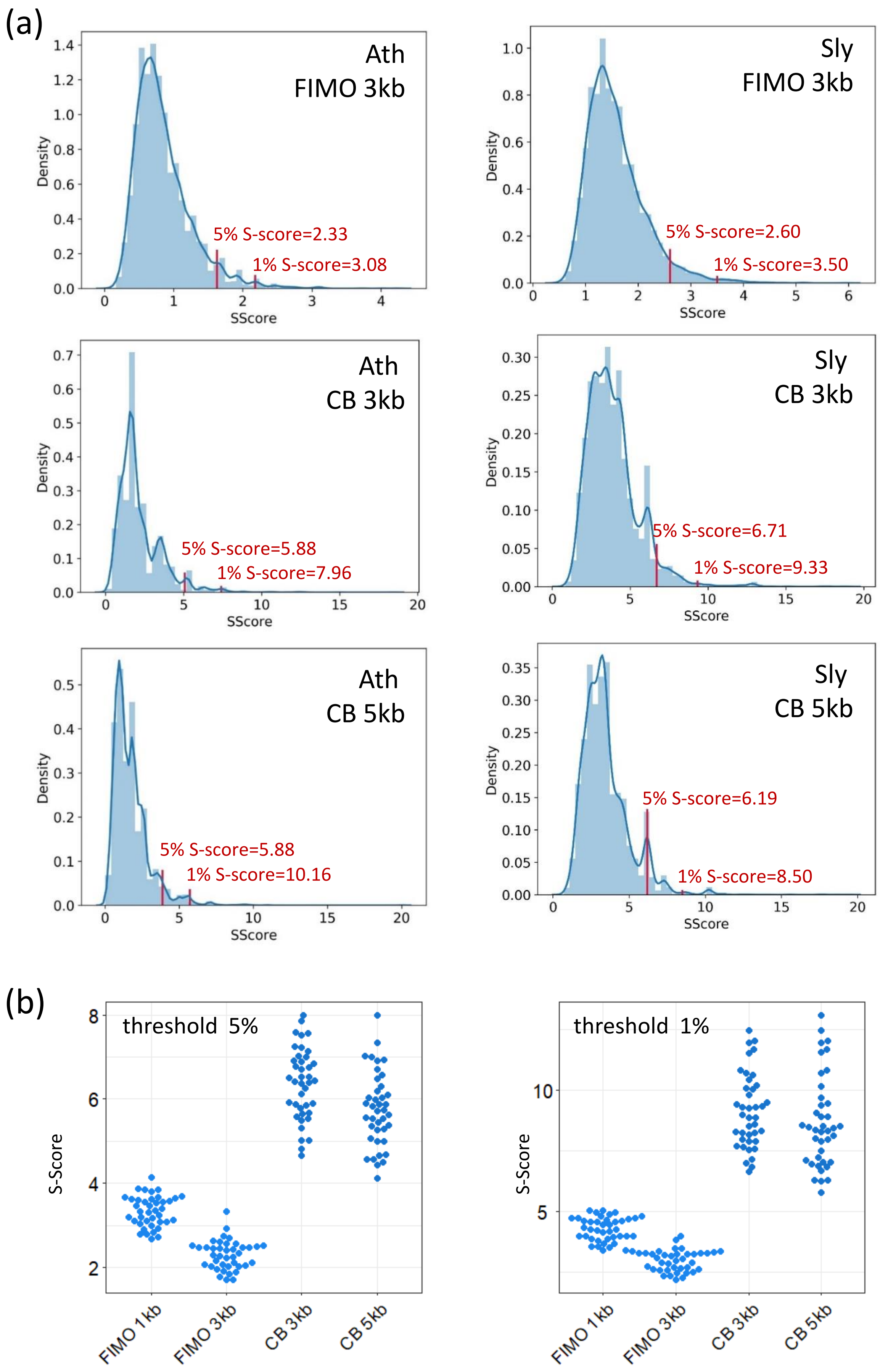

Figure S3. (a) Density plots of the significance score (S-Score) generated for estimation of the 5% and 1% threshold S-Scores as false positive evaluation. Red lines represent the S-Score values for the top 1% and 5% thresholds of the distribution. Plots correspond S-Scores evaluation in Arabidopsis data (left) and tomato (right). (b) Distribution of threshold S-Scores of the 40 species at 5% (left) and 1% (right).

Figure S4

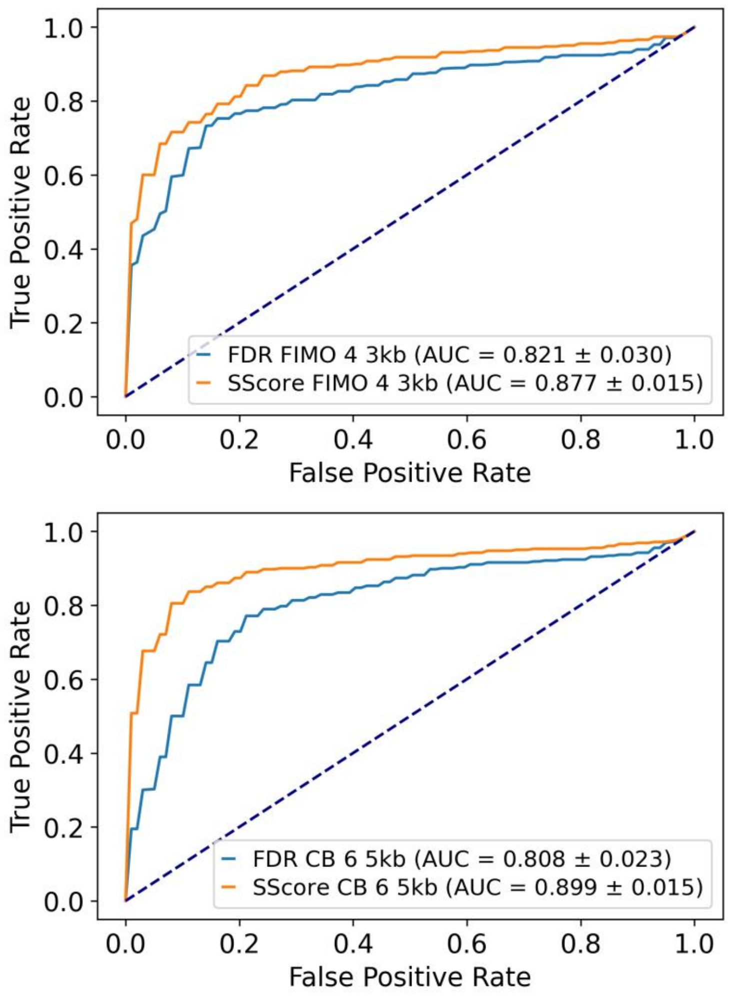

Figure S4. ROC curves and AUC values for estimation of the best filtering values, S-Score and FDR. Plots correspond to two searches launched with the parameters indicated.
